# Spatio-temporal dynamics of urban medical system carrying capacity and their obstacle factors: A case study of Yangtze River Delta urban agglomeration

**DOI:** 10.1371/journal.pone.0319638

**Published:** 2025-04-09

**Authors:** Chengshuang Sun, Ke Zhou, Guangxia Li, Weina Zhu, Dongjun Wan

**Affiliations:** School of Economics and Management Engineering, Beijing University of Civil Engineering and Architecture, Beijing, China; Huazhong University of Science and Technology, CHINA

## Abstract

The frequent occurrence of various urban disasters poses risks to human survival and welfare, and it is of great significance to evaluate the urban medical system carrying capacity (UMSCC), monitor the spatial and temporal patterns of the UMSCC and identify their obstacle factors to improve the public medical system and rationalize medical resource allocation. In this paper, the Yangtze River Delta urban agglomeration (YRDUA), which includes 27 major cities, is taken as a case study. A UMSCC evaluation model is constructed and the gravity center shift trajectory is analyzed by using the ArcGIS software. The results indicate that the UMSCC of the 27 cities in the YRDUA can be classified into 5 levels: the lowers (0.193-0.335), the lows (0.335-0.425), the mediums (0.425-0.489), the highs (0.489-0.549) and the highers (0.549-0.619). From 2011-2021, the UMSCC level in all the 27 cities from the YRDUA increased annually, and during this period, the gravity center of the UMSCC was concentrated in Xuancheng, and the migration trend was southwest. Moreover, there is a positive correlation between the city type and the level of UMSCC: the larger of the city is, the higher of the UMSCC level is; however, the gap between the levels of UMSCC in different city types in the YRDUA gradually narrows and tends to be consistent, which reflects the development trend of medical system integration in the region. Finally, it is concluded that the key obstacle indicators of the UMSCC in the YRDUA can be attributed to the proportion of medical financial expenditure, the number of beds, the number of registered nurses and the urban digital development level. Correspondingly, the suggestions are proposed.

## Introduction

With the accelerated urbanization and increasingly urban populations, the demand for public health services has increased dramatically. According to relevant reports from the World Health Organization, 90% of countries have experienced disruptions in basic health services since the Corona Virus Disease 2019 (COVID-19) [1]. To cope with population expansion and sudden public health crises, improving the public health system and rationalizing the allocation of medical resources have attracted the attention of governments around the world. The United Nations 2023 Agenda for Sustainable Development indicates that cities are at the front line of the fight against pandemics and their continuing impacts. Therefore, research on medical system carrying capacity is of great significance for the prevention and control of new pandemics and the construction of disaster-resistant cities in the future. Wu et al. [2] evaluated the carrying capacity of medical resources in Shenzhen from the perspective of supply and demand, and provide reference for planning of medical resources and improving the carrying capacity of medical resources. Wu et al. [3] analyzed the carrying capacity of public medical resources in Shenzhen, and provided basis the allocation of medical resources in mega-city. Emanuel et al. [4] studied the distribution of medical resources in the United States, South Korea, the United Kingdom and other countries in the time of COVID-19 to help solve how to allocate medical resources fairly. However, these studies mainly focused on some specific city or nation, which is lack of the exploration on urban agglomerations.

In 2014, the Chinese government issued the “Notice on Adjusting the Classification Standards for the Size of Cities” document, which divides cities into seven levels according to the permanent resident population, namely, Type I small cities, Type II small cities, Medium-sized cities, Type I large cities, Type II large cities, Super-cities, Mega-cities. Different types of cities present various problems in the UMSCC. For example, since the mega-cities have accumulated experience in responding to major public health events [5], the capacity for comprehensive disease prevention and control as well as the medical infrastructure level have greatly improved. However, the mega-cities still face some problems, such as mismatches between the scale of facilities and the growth of demand, and imbalances in resource supply [6]. In addition, there is a gap in medical services levels between the small & medium-sized cities and large cities. The efficiency and equity of medical service resource allocation has always affected the development of small and medium-sized cities [7].

As for the assessment of urban carrying capacity, scholars have proposed a variety of methods based on different research objects, including mean variance analysis [8,9], hierarchical analysis [10,11], the entropy method and data envelopment analysis [12], the multi-objective linear summation method [13], the “load-carrier” model [14], etc. Among them, the “load-carrier” model has a wider application. Shen et al. [14] applied the “load-carrier” model to evaluate the carrying capacity of urban resources and environment, revealing the dynamics, state and bottoming of the carrying capacity; then, their team continued to study the carrying capacity of the ecological environment [15] and municipal infrastructure carrying capacity [16] to help policymakers design policies to increase the carrying capacity. Cui et al. [17] developed a new assessment method for evaluating the economic carrying capacity of urban infrastructures from the perspective of loads and carriers, combining the entropy weight method, the linear weighted sum method, and measurement model based on meta-boundary relaxation. Based on the research above, this paper adopts the “load-carrier” model to study medical system carrying capacity.

Therefore, from the perspective of time and space, the medical system carrying capacity in different cities of the YRDUA is quantitatively evaluated. This paper analyzes the evolution law and spatial distribution of the UMSCC, explores the relationship between different city types and different levels of the UMSCC, and identifies the key constrained indicators for improving medical carrying capacity. In addition, this paper combines the mean variance analysis and the coupling coordination degree model, and applies the “load-carrier” model to study the medical system carrying capacity of 27 major cities in the YRDUA of China from the perspective of spatio-temporal evolution, and then provides countermeasures and suggestions to improve the public medical system and rationalize medical resource allocation. These findings provide a reference for the Chinese government and other developing countries to formulate measures to improve the UMSCC, promote coordinated development of the regional medical system, and improve people’s well-being in China.

Compared with previous studies, the innovation of this paper is mainly reflected in the following three aspects. First, most of the previous studies on medical resources focused on some specific cities and lacked research on urban agglomerations. And, with the outbreak of COVID-19, medical research has attracted widespread public and academic attention. These studies are usually conducted from the perspective of virus development itself, and there is a lack of the medical resources analysis from the perspective of urban agglomerations. This paper focuses on the construction and improvement of urban agglomeration public health emergency response center. Second, the existing studies related to medical resources tend to focus on the accessibility [18] and equity [19] of medical resources in some cities, yet few studies explore the supply and demand relationship of medical resources and the evaluation of the carrying capacity of medical resources in the urban agglomeration. Third, previous scholars used obstacle degree model to identify obstacle factors, which was greatly affected by weight and could not directly and objectively reflect the obstacle factors. This paper makes reference to the national policy documents when studying the obstacle factors. By comparing the standard of each index in the document with the actual development situation of the city, we can find out which aspect of the city still has shortcomings, and put forward policy suggestions for the UMSCC improvement by analyzing this deficiency. This method is targeted and can analyze the defects of the carrying capacity of each city’s medical system from the actual development situation. Therefore, this paper analyzes the UMSCC of the Yangtze River Delta to improve the regional medical resource integration and sharing, which is conducive to the development of the Yangtze River Delta region and has practical significance for the integrated development of urban agglomerations in China.

## Research framework

**Fig 1:**
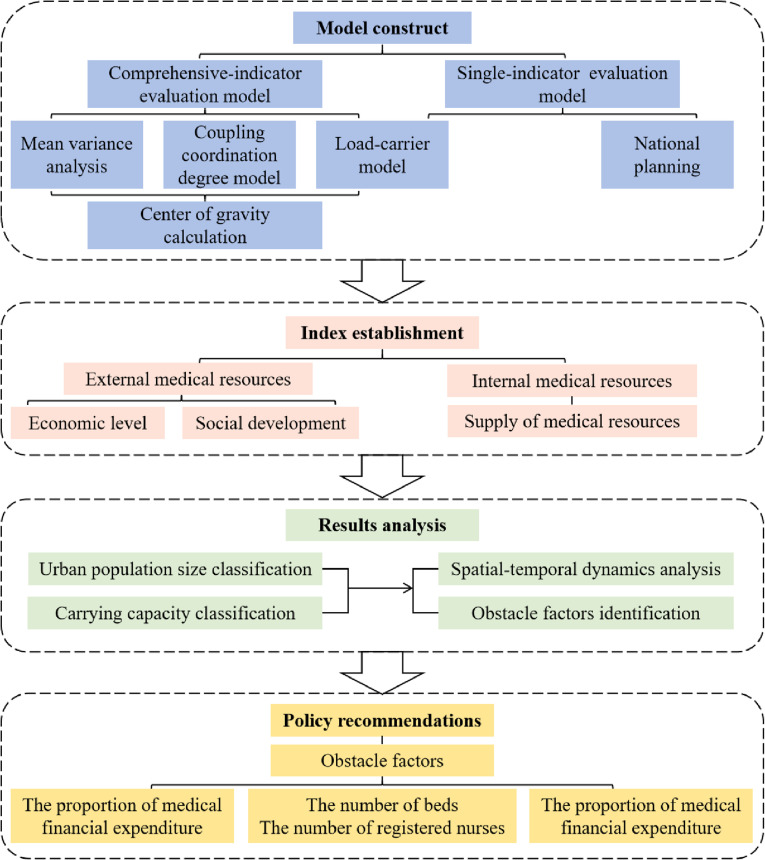
Research framework of this study.

The research framework of this paper is shown in Fig 1. First, an evaluation model of the UMSCC is constructed, which is mainly divided into single-indicator evaluation model and comprehensive-indicator evaluation model. Second, the evaluation index system for the UMSCC is established. The evaluation index system of the urban medical system is composed of three dimensions: economic level, social development and medical resource supply level. And then, based on the above evaluation index system and evaluation model, the UMSCC from the perspective of spatio-temporal evolution is analyzed and the obstacle factors are identified. Finally, on the basis of the key obstacle factors, some suggestions are put forward for improving the public health system and rationalizing medical resource allocation.

## Materials and methods

### Study area

**Fig 2:**
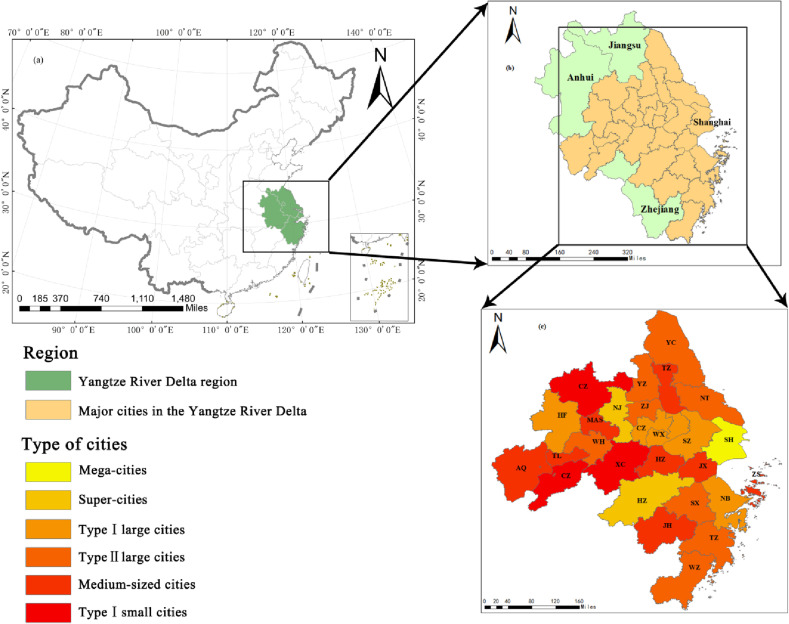
Overview map of the study area. **(a)** Location of the YRDUA region in China; **(b)** Overview of the YRDUA region; **(c)** 27 major cities in the YRDUA region; Classification of cities by population size. These figures were created by the authors using a shapefile of the administrative division of China, which is publicly available on the government’s “National Geographic Information Public Service Platform of China” (Open data in China) web page at https://www.tianditu.gov.cn. Population data are shown in Appendix 1.

The Yangtze River Delta region is one of the most economically active and open regions in China, and the integrated development of the Yangtze River Delta region has been elevated to a national strategy by the government. According to the “Outline of the Plan for the Integrated Development of the Yangtze River Delta Region” issued by the Central Committee of the Communist Party of China and the State Council, it is determined that the scope of the plan includes the entire area of Shanghai, Jiangsu Province, Zhejiang Province and Anhui Province, as shown in Figs 2 (a) and (b). The outline stipulates that 27 cities, including Shanghai, Nanjing, Wuxi, Changzhou, Suzhou, Nantong, Yangzhou, Zhenjiang, Yancheng, Taizhou, Hangzhou, Ningbo, Wenzhou, Huzhou, Jiaxing, Shaoxing, Jinhua, Zhoushan, Taizhou, Hefei, Wuhu, Ma ‘anshan, Tongling, Anqing, Chuzhou, Chizhou and Xuancheng, are the central areas, as shown in Fig 2(c). The central areas, namely the 27 cities, can be the representative of the YRDUA to explore the UMSCC in this paper. At present, there is a large gap in the scale and quality of medical services in the YRDUA. For example, according to statistics, the number of hospitals per million people in Nanjing in 2021 was 29.4; Shanghai, although the only first-tier city in the YRDUA, only was 17.4 in the number of hospitals per million people.

The 27 major cities in China’s YRDUA can be divided into 6 categories according to the number of permanent residents, namely, mega-cities, super-cities, type I large cities, type II large cities, medium-sized cities and type I small cities, as shown in Fig 2(c).

### Carrying capacity evaluation model

#### The “load-carrier” model.

The core of the UMSCC is the ability to match the supply of medical services with the demand of residents for medical services. Therefore, it is appropriate to use the “load-carrier” model to study the construction of the indicator system in the dimension of medical resource supply. Based on the research of Shen et al. [14] on resource and environment carrying capacity, it can be seen that the UMSCC is a dynamic equilibrium state maintained under the interaction between medical load and medical carrier. The above analysis shows that the UMSCC under the “load-carrier” model reflects the extent to which medical resources can carry the medical load. That is the degree to which the resources respond to the pressure exerted by the medical needs of the population. The expression for the UMSCC based on the “load-carrier” relationship is shown in (1):


$$X=\frac{L}{C}$$ (1)

Where *X* denotes the actual value used to reflect the indicator, load (*L*) refers to the various pressures from the health needs of the population; and carrier (*C*) refers to the supporting force that can meet the health needs of the population. The methodology serves as an innovative evaluation framework for the UMSCC, integrating loads and carriers, as well as the interactions between them.

The load acts on the carrier using exerting pressure, and the degree of exerted pressure is mainly influenced by human age structure, health needs, and other factors, including conventional load and dynamic load, which are not considered in this paper due to research limitations. The carrier is the key factor to support the production and life of residents. The medical carriers in this model refer to internal medical resources, which are related to the human and material resources needed for health services.

### Comprehensive-indicator carrying capacity evaluation model

This paper combines the mean variance analysis and the coupling coordination degree model to establish a UMSCC evaluation model. Compared with other multi-indicator comprehensive evaluation methods, the mean variance analysis is more objective and avoids the subjective influence of researchers. It can synthesize and integrate a large number of variables so that the results are more scientific and accurate. Currently, the mean variance analysis has been used for social carrying capacity [20], natural resource carrying capacity [21], comprehensive urban carrying capacity [8], etc. The coupling coordination degree model is a method used to study the coupling, coordination and feedback of systems and internal constituent variables, which has been used to evaluate urban infrastructure carrying capacity [22]. The combined use of these two models considers not only the integration of multiple variables but also the role of coupling and coordination between multiple variables.

Taking the economic level dimension as an example, the mathematical model used to analyze the UMSCC is given by equations (2)-(4) [8]:


$$F_{1}=\sqrt{U_{1}\times V_{1}}$$ (2)

$$U_{1}=n\frac{\sqrt[{n}]{\prod {}_{i=1}^{n}Y^{'}{}_{i}}}{\sum {}_{i=1}^{n}Y^{'}{}_{i}}$$ (3)

$$V_{1}=\frac{1}{n}\sum {}_{i=1}^{n}\left\{Y^{'}{}_{i}\sqrt{{\left[Y^{'}{}_{i}-\frac{1}{n}\sum {}_{i=1}^{n}Y^{'}{}_{i}\right]}^{2}/\sum {}_{i=1}^{n}{\left[Y^{'}{}_{i}-\frac{1}{n}\sum {}_{i=1}^{n}Y^{'}{}_{i}\right]}^{2}}\right\}$$ (4)

Where *F*_*1*_ denotes the carrying capacity provided by the economic level dimension, *U*_*1*_ denotes the value of the coupling coordination degree of the indicators reflecting the economic level dimension, *V*_*1*_ denotes the value of the combined integration of the indicators reflecting the economic level dimension, *Y’i* denotes the standardized value of the *ith* evaluation indicator used to reflect *F*_*1*_, and *n* denotes the number of evaluation indicators used to reflect *F*_*1*_.

Since the dimensions of the data collected in this paper are not uniform, it is necessary to normalize these original data to eliminate the obstacles to data analysis caused by the disunity of units. This study uses the range method to address dimensional changes, in which the positive indicators are processed by the equation (5), and the negative indicators are processed by the equation (6).


$$Y^{'}{}_{i,\;j}=\frac{Y_{i,j}-\min(Y_{1,j},Y_{2,j}\cdots Y_{m,j})}{\max(Y_{1,j},Y_{2,j}\cdots Y_{m,j})-\min(Y_{1,j},Y_{2,j}\cdots Y_{m,j})}$$ (5)

$$Y^{'}{}_{i,\;j}=\frac{\max(Y_{1,j},Y_{2,j}\cdots Y_{m,j})-Y_{i,j}}{\max(Y_{1,j},Y_{2,j}\cdots Y_{m,j})-\min(Y_{1,j},Y_{2,j}\cdots Y_{m,j})}$$ (6)

Taking the economic level dimension as an example, *Y*’*i,j* denotes the standardized value of the *ith* evaluation indicator for region *j*, *Y*_*i,j*_ denotes the original value of the *ith* evaluation indicator for region *j*, max(*Y*_*i,1*_*, Y*_*i,2*_ *··· Y*_*i,m*_) denotes the maximum value of indicator *i* among the *m* study regions; min(*Y*_*i,1*_*, Y*_*i,2*_ *··· Y*_*i,m*_) denotes the minimum value of indicator *i* among the *m* study regions.

The UMSCC is supported by a combination of economic, social, and healthcare resources, so the final expression for the UMSCC is shown in (7).


$$F=\frac{F_{1}+F_{2}+F_{3}}{3}$$ (7)

### Center of gravity calculation

The center of gravity is mainly used to describe the spatio-temporal change process and development trend of a certain index. In physics, it refers to the point at which the resultant forces of various parts of an object are balanced. If the center of gravity shifts, it means that the resultant force changes and a new equilibrium point is formed. The center of gravity position of the medical system carrying capacity of the Yangtze River Delta is the equilibrium point of the mass resultant force of the particles of the medical system carrying capacity of each city. The calculation formula of the medical system carrying capacity center of gravity is as follows.


$$X=\frac{\sum_{j=1}^{n}X_{j}F_{j}}{\sum_{j=1}^{n}F_{j}},Y=\frac{\sum_{j=1}^{n}Y_{j}F_{j}}{\sum_{j=1}^{n}F_{j}}$$ (8)

In the formula, *X*_*j*_ and *Y*_*j*_ respectively represent the longitude and latitude of the geometric center of city *j*, *F*_*j*_ represents the carrying capacity of the medical system of city *j*, and *X* and *Y* represent the longitude and latitude of the carrying center of the medical system.

### Single-indicator carrying capacity evaluation model

In this paper, we construct the single-indicator medical system carrying capacity equations as (9)-(10) [23]:


$$Y=Y_{i}-Y_{t}$$ (9)

$$M=\frac{Y}{Y_{t}}$$ (10)

The economic level dimension is taken as an example, where *Y* denotes the amount of overload, *Y*_*i*_ denotes the actual value, *Y*_*t*_ denotes the critical value of overload, and *M* denotes the overload rate. When the indicator is positive, a *Y* value greater than 0 indicates an overloaded state, and a *Y* value less than 0 indicates the existence of remaining carrying space. When the indicator is negative, *Y* has the opposite meaning as described earlier.

## Indicator construction and data collection

### Indicator construction

In this paper, the index of the UMSCC is established from two perspective: internal influencing factors and external influencing factors. The “load-carrier” model is used to identify the internal influencing factors, which includes load and carrier indicators. The load refers to the demand generated by the population, which creates pressure on the carrier. The carrier refers to the supply of internal medical resources to meet the demand. Meanwhile, the external (macro) social and economic development factors related into medical resources can also influence the UMSCC, mainly including proportion of medical financial expenditure, urban digital development index and so forth.

Specifically, the urban medical service system, on the one hand, has indispensable resources to carry the load and guarantee the normal operation of the urban medical service, including the level of basic medical staffing, the level of medical technology, and medical supplies. On the other hand, the normal operation of medical services and their adaptability in the face of sudden shocks cannot be separated from the support of various external factors, such as the local economic situation, the degree of digitization, the level of urbanization, and so on. These factors are related to the social, economic, and medical resources themselves, which are the pillars of the sustainable development of health care. Therefore, the three aspects of economic level, social development, and medical resource supply are used as guidelines for the selection of evaluation indicators [ 33–35].

Financial investment in medical care affects medical resources, which in turn affects access to and utilization of medical services, the quality of medical services, and the quality of medical services [25,36–38]. The per capita disposable income can reflect the income level of local residents and their ability to pay for medical services, and some scholars have deemed that the increase in income has a more obvious effect on the level of medical resource allocation [39–42]. The level of urbanization reflects the comprehensive strength of the city and affects the allocation and utilization of medical resources [43,44]. Basic medical insurance, as a bottom-up tool to compensate patients for economic losses due to illness, is closely related to the local economic level, medical resources, and consumption level of residents [45–49]. The number of beds, as a commonly used indicator for measuring medical facilities or physical resources, constitutes one of the necessary foundations for the effective operation of medical services [50–52]. Medical human resources have a significant impact on medical outcomes and are considered central to the proper functioning of medical services [53–55]. The load (L) studied in this paper is mainly conventional load, which refers to the basic healthy living needs of residents. It is specifically embodied in the residents’ demand for preventive health care, disease treatment, rehabilitation services, health education, emergency relief and so on. The pressure of medical carrying comes from urban residents, so the regional resident population [17] is used as a load indicator to study the UMSCC. This reflects the scientific and rigorous nature of the study.

This paper first uses literature analysis to search the Web of Science database and screen out relevant indicators. In addition, the relevant policy documents are combined in this paper to refine the indicators construction, such as the 14th Five-Year National Information Plan, the 14th Five-Year Plan for National Economic and Social Development of the People’s Republic of China, and the Outline of the 2035 Vision Goals, all of which strengthen the future of China’s health care’s importance, and thus combining these plans to revise theses indicators is conducive to effectively exploring the current carrying capacity of the Yangtze River Delta medical system and planning the future medical development of the Yangtze River Delta. Also, the interviews with experts in related fields are conducted to amend further, and finally the carrying capacity evaluation index of the urban medical system is obtained, as shown in Table 1.

**Table 1:** Indicators for evaluating the UMSCC.

Target level	Criterion layer	Sub-criteria layer		Indicator layer	Literature
Indicators for evaluating medical system carrying capacity	External medical resources	Economic level	Proportion of medical financial expenditure (Y_1_)		[24,25]
			Disposable income per capita (Y_2_)		[26,27]
		Social development	Urban Digital Development Index (Y_3_)		[28]
			Level of urbanization (Y_4_)		[26,29]
			Basic health insurance participation rate (Y_5_)		[30,31]
	Internal medical resources	Supply of medical resources	Carrier indicators	Number of beds (C_1_)	[2,24,32]
				Number of practicing (assistant) physicians (C_2_)	[2,32]
				Number of registered nurses (C_3_)	[2,32]
				Number of hospitals (C_4_)	[2,24]
			Load indicators	Number of resident urban population (L_1_)	[2]

### Data collection

The original data of 8 indicators in this paper is collected from the Statistical Yearbooks (2011-2021) of 27 cities in this paper and also the Statistical Bulletins of National Economic and Social Development are used to supplemented some missing data. The data of urban digital development index is gathered from the “Urban Digital Development Index” (http://deindex.h3c.com) published by H3C Technologies Company. A small amount of missing annual data was supplemented according to the index smoothing method. The empirically calculated data for this study were collected from 27 major cities in the YRDUA of China from 2011-2021.

## Results and analysis

### Time evolution of the UMSCC

Equations (1)-(7) were used to calculate the average medical system carrying capacity in 27 major cities in the YRDUA, and the evaluation results from 2011-2021 are shown in Table 2.

**Table 2:** Measured average values for each sub-dimension of the UMSCC, 2011-2021.

Year Dimension	2011	2012	2013	2014	2015	2016	2017	2018	2019	2020	2021	$\bar{x}$	S^2^
Economic level	0.109	0.124	0.134	0.147	0.154	0.158	0.164	0.167	0.172	0.180	0.188	0.154	0.0006
Social development	0.136	0.143	0.148	0.150	0.150	0.153	0.156	0.162	0.164	0.164	0.167	0.154	0.0001
Supply of medical resources	0.142	0.159	0.167	0.173	0.176	0.184	0.186	0.193	0.198	0.203	0.203	0.180	0.0004
Combined carrying capacity	0.387	0.426	0.449	0.470	0.480	0.495	0.505	0.522	0.534	0.548	0.557	0.488	0.0028

During the study period, the average value of the comprehensive carrying capacity of the medical system and the average value of each sub-dimension of the 27 major cities showed an upward trend. In addition, it can be seen that the urban medical system has a relatively high carrying capacity for the medical resource supply dimension. The carrying capacity of the economic level dimension has changed considerably, from 0.109 to 0.188. This indicates that the economic level of the Yangtze River Delta cities developed relatively fast from 2011-2021, which is mainly reflected in the overall rising trend of per capita disposable income during these 11 years.

### Analysis of the UMSCC level in different city types

**Fig 3:**
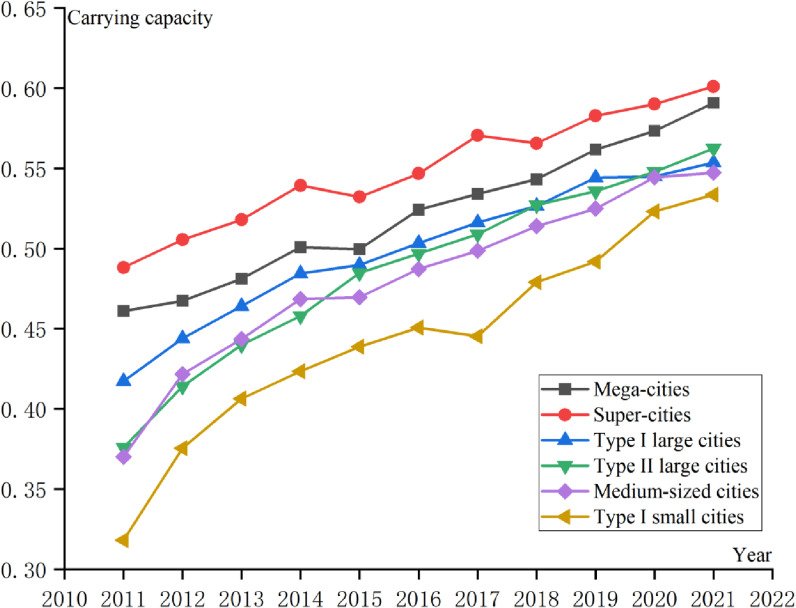
Development trend of the UMSCC of the 6 types of cities in the YRDUA.

The UMSCC from 2011-2021 was studied according to the type of city (Fig 3):

(1) Over time, the medical system carrying capacity of the six types of cities gradually increased. This indicates that from 2011-2021, the economic level, social development and medical resource supply level of cities significantly improved. The growth rate of Type I small cities is the fastest, which shows that the Chinese government has realized that small cities are an important growth point of national strength in the process of building a modern socialist country, so it attaches great importance to the development planning of small cities.
(2) The carrying capacity of super-cities is the highest, and the carrying capacity of Type I small cities is the lowest. This indicates that the UMSCC is closely related to the comprehensive strength of the city. Cities with greater comprehensive strength can provide greater economic support for the medical system, have a higher level of digitalization, attract more medical talent, and possess a higher level of medical configuration, so the medical system carrying capacity is greater. The opposite is true for cities with lower overall strength.
(3) The gap between the medical system carrying capacity in the six types of cities is gradually narrowing as illustrated in Fig 3. This shows that no matter the size of the urban population in the future, there will not be a large gap in the carrying capacity of the medical system between cities. In 2018, the Chinese government elevated the development of regional integration in the Yangtze River Delta to a national strategy, and medical integration was one of the priorities of this strategy. The development of medical integration is conducive to the cooperation of three provinces and one city to establish a medical system, implement a unified basic medical insurance policy, and enjoy the same medical services. In addition, this also reflects the Chinese government’s steady improvement in people’s livelihood over the course of development.

### Analysis of the UMSCC level in 27 cities

**Fig 4:**
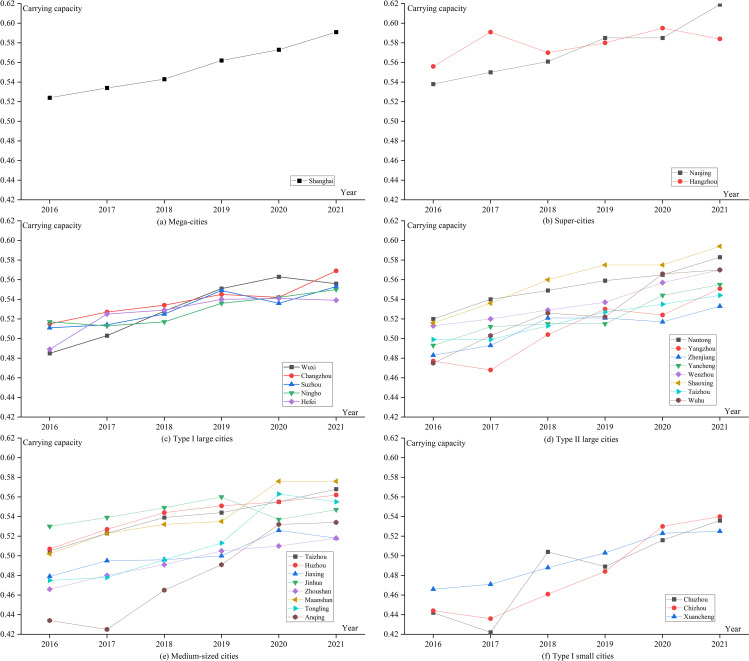
Trends in the UMSCC of 27 major cities in the YRDUA.

To further study the detailed and up-to-date changes during the 13th Five-Year Plan period from the perspective of 27 major cities, this study analyzes the medical system carrying capacity from 2016-2021 based on the 6 types of cities (Fig 4):

[1] Mega-cities and Super-cities have the highest carrying capacity of the medical system. Type I small cities have the lowest carrying capacity of the medical system, but are growing faster, as shown in Figs 4 (a)、(b) and (f). Shanghai is at the core of the YRDUA, whereas Nanjing and Hangzhou have the status of the YRDUA sub-centers. These three cities have the highest carrying capacity of the medical system, as they have gathered a large number of high-quality medical resources, a high-quality economy and a high level of social development that far exceeds that of other cities. Type I small cities have fewer industries, backward economic development, and insufficient allocation of medical resources. However, under the guidance of the Yangtze River Delta integration strategy, high-quality medical resources in Jiangsu, Zhejiang, and Shanghai have been extended and radiated there. Moreover, the government has accelerated the integration and development of emerging information technology and the healthcare industry, which has comprehensively improved the level of medical technology services.
[2] The gap in medical system carrying capacity among cities in Type I large cities is small, and the gap in medical system carrying capacity among cities in medium-sized cities is large, as shown in Figs 4 (c) and (e). The development of each city in Type I large cities is balanced, and the gap in medical system carrying capacity is small. There is a gap in the medical system carrying capacity of each city in medium-sized cities, such us Anqing and Taizhou. The medical system carrying capacity in Anqing has undergone a large change, showing a decreasing and then increasing trend from 2016-2021, with a decrease in 2017 and a faster growth from 2018-2020. The medical system carrying capacity in Taizhou has shown a steady upward trend.
[3] Only Type II large cities and Type I small cities have an overall upward trend in medical system carrying capacity, as shown in Figs 4 (d) and (f). There is a downward trend in the medical system carrying capacity of the remaining city types, such as Hangzhou and Wuxi. Hangzhou, the capital of Zhejiang Province, has experienced population growth with high-quality economic development, attracting people from other cities. This has led to excessive urban pressure and an uneven distribution of medical resources.

### Spatial distribution and dynamics of the UMSCC

#### Spatial distributions of the different carrying capacity classifications.

**Fig 5:**
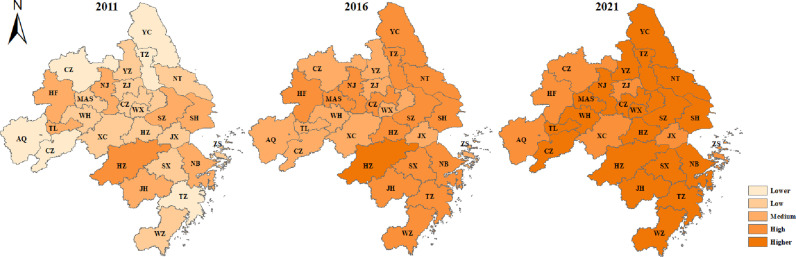
The UMSCC in 27 major cities of the YRDUA in 2011, 2016 and 2021. These figures were created by the authors using a shapefile of the administrative division of China, which is publicly available on the government’s “National Geographic Information Public Service Platform of China” (Open data in China) web page at https://www.tianditu.gov.cn. The city name of abbreviation and raw data for these 27 cities are shown in Appendix 1 and 2.

To further compare the differences and the evolution in the spatial patterns during the different time units of medical system carrying capacity in cities within the YRDUA, the carrying capacity was categorized into the lower carrying capacity zone (0.193-0.335), the low carrying capacity zone (0.335-0.425), the medium carrying capacity zone (0.425-0.489), the high carrying capacity zone (0.489-0.549), and the higher carrying capacity zone (0.549-0.619), by using the natural breakpoint method. The spatial distribution of medical system carrying capacity in 27 major cities in the YRDUA was mapped via ArcGIS software, as shown in Fig 5.

The 2011, 2016 and 2021 data represent the carrying capacity of 27 major cities in the YRDUA of China at the beginning of the 12th, 13th and 14th Five-Year Plan, respectively. As can be seen in Fig 5, the findings are as followings, (1) in 2011, among the 27 cities, the cities with low and medium carrying capacity were the most numerous, and they were mainly distributed in the central region; (2) compared with medical system carrying capacity in 2011, the medical system carrying capacity in 2016 improved to a certain extent, with the disappearance of cities with low and lower carrying capacity, and the cities with medium and high carrying capacity being the main ones, and the southern region’s carrying capacity was significantly better than that of the northern region; (3) relative to 2016, 2021 was dominated by high carrying capacity and higher carrying capacity cities, with inland cities having significantly lower carrying capacity than coastal cities.

Overall, the medical system carrying capacity of 27 major cities in the YRDUA of China has trended to increase over the past 11 years. This is mainly attributed into the fact that the integrated development of healthcare in the Yangtze River Delta has enabled the region to realize a deep integration of healthcare management, technology, services, and research, and to build a good situation of healthcare development, public benefits, and win-win cooperation.

### Gravity center migration analysis of the UMSCC

**Fig 6:**
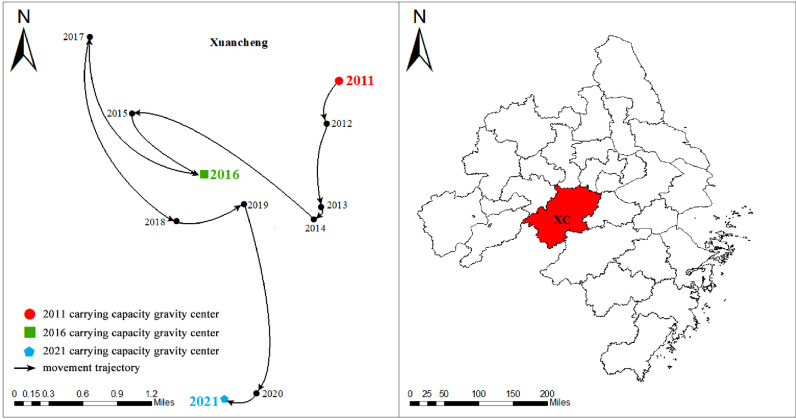
The UMSCC gravity center shift trajectory.

The center of gravity of the UMSCC was calculated according to formula (8). The movement trajectory of the gravity center was analyzed, and the overall trend was found to be southwest, as shown in Fig 6. From 2011-2016, the center of gravity shifted 2.14 kilometers to the southwest; from 2016-2021, the center of gravity moved 3.19 kilometers to the southeast. This shows that the growth rate of the medical system carrying capacity in the southern cities of the Yangtze River Delta is higher than the average level, and the regions with the highest growth rate of the overall medical system carrying capacity continue to expand to the south. It can be seen from Table 3 that from 2011-2021, the longitude of the center of gravity of the UMSCC mainly varied between 119° 56’ 17 “E and 119° 59’ 33” E, and the latitude mainly varied ranging from 31° 00’ 27 “N to 31° 04’ 87” N, and the center of gravity was always located in Xuancheng city in the middle of the Yangtze River Delta. This indicates that cities with high carrying capacity were concentrated around the city during this period, which is consistent with the UMSCC shown in Fig 5.

**Table 3:** Gravity center shift condition.

Year	Longitude	Latitude	Shift of Gravity Center	
			Orientations	Length/km
2011	119°59’33’‘E	31°04’31’‘N		
2012	119°59’17’‘E	31°03’77’‘N	southwest	0.62
2013	119°59’10’‘E	31°02’71’‘N	southwest	1.18
2014	119°59’01’‘E	31°02’56’‘N	southwest	0.19
2015	119°56’70’‘E	31°03’89’‘N	northwest	2.65
2016	119°57’54’‘E	31°03’14’‘N	southeast	1.16
2017	119°56’17’‘E	31°04’87’‘N	northwest	2.32
2018	119°57’27’‘E	31°02’53’‘N	southeast	2.79
2019	119°58’12’‘E	31°02’75’‘N	southeast	2.61
2020	119°58’28’‘E	31°00’35’‘N	northwest	2.66
2021	119°57’87’‘E	31°00’27’‘N	southwest	2.75

### Analysis of the relationships between the UMSCC and city type

**Fig 7:**
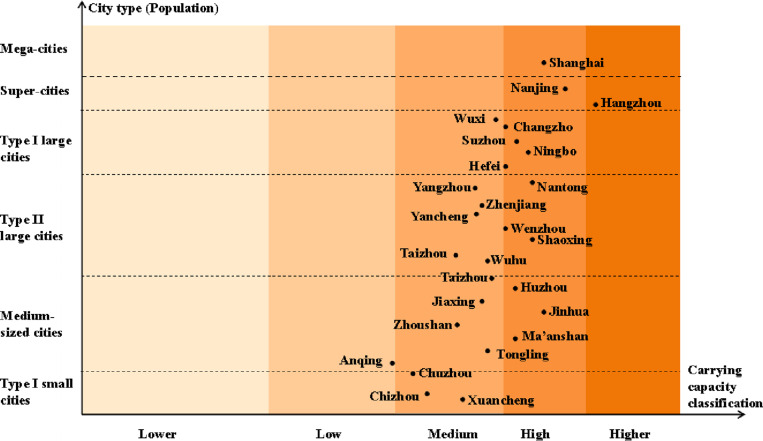
Relationships between the average UMSCC and the 6 types of cities in the YRDUA.

Based on the analysis of the average carrying capacity of the medical system of each city from 2011-2021 by combining the city type and carrying capacity zone, it can be concluded that as shown in the Fig 7:

(1) The UMSCC in 27 major cities of the Yangtze River Delta is mainly distributed in the medium carrying capacity zone and the high carrying capacity zone. This indicates that the comprehensive level of medical system carrying capacity in the Yangtze River Delta region is relatively good. Moreover, as shown in Fig 7, all the medical system carrying capacity levels from each city are distributed more evenly along one line, indicating that the UMSCC is positively correlated with the size of the urban population.
(2) As the only mega-city in the YRDUA, Shanghai’s medical system is located in a high carrying capacity zone. According to studies by other scholars on the carrying capacity of medical systems in mega-cities, the medical supply level in mega-cities is slightly higher than the demand level [2]. This shows that the current medical system in China’s mega-cities has good carrying capacity and can basically meet the needs of residents.
(3) Hangzhou’s medical system has the highest carrying capacity and is located in the higher carrying capacity zone, which indicates that Hangzhou pays more attention to residents’ medical services. Anqing’s medical system has the lowest carrying capacity, which affects the overall carrying capacity of the Yangtze River Delta region. This shows that there are still difficulties in the development of small cities in China, and the Chinese government needs to focus on such cities.

### Identification and analysis of obstacles to improving the UMSCC

In this paper, China’s latest development goal planning of relevant indicators is taken as the critical value of overload, as shown in Table 4. Since the number of hospitals is not currently planned, the other 8 indicators were evaluated. Due to missing data for some of the indicators for 2011-2016, this will have an impact on the results. Therefore, the years 2017-2021 were selected for comparative analysis. The state of the worst indicator of the 8 indicators is taken as an obstacle factor of the UMSCC.

**Table 4:** Criteria for classifying the evaluation indicators of the UMSCC.

Indicator	Overload threshold	Reference point	No overload	Overload
Proportion of financial expenditure on medical care (A)	11%	Research report on the “Healthy China 2020” strategy	Y ≥ Y_t_	Y < Y_t_
Basic health insurance participation rate (B)	95%	China’s 14th Five-Year Plan for Universal Healthcare Coverage		
Urban digital development index (C)	95	China’s 14th Five-Year for National Information Plan		
Level of urbanization (D)	65%	China’s 14th Five-Year Plan		
Growth in per capita disposable income (E)	2.10%			
Number of beds (F)	0.018 million	Guiding Principles for the Establishment and Planning of Medical Institutions in China (2021-2025)	X ≤ X_t_	X> X_t_
Number of practicing (assistant) physicians (G)	0.031 million			
Number of registered nurses (H)	0.026 million			

According to formulas (9) - (10) and Table 4, the overloading situation of each city is calculated, and the index with the highest overloading rate (%) of each city is selected as the obstacle factor of the UMSCC. As shown in Table 5, the obstacles to the UMSCC in the Yangtze River Delta in 2021 mainly focus on the proportion of medical financial expenditure (A), the urban digital development index (C), the number of beds (F), and the number of registered nurses (H).

**Table 5:** Comparison of single-indicator overloads for the 27 cities from 2017-2021.

City	Year									
	2021		2020		2019		2018		2017	
	Indicator	Over-load rate	Indicator	Over-load rate	Indicator	Over-load rate	Indicator	Over-load rate	Indicator	Over-load rate
Jiaxing	A	40.01	A	42.00	A	51.64	A	44.64	A	40.45
Hefei	A	39.73	A	32.36	A	38.64	A	35.55	A	29.00
Suzhou	A	39.09	A	45.64	A	45.64	A	47.45	A	46.91
Zhenjiang	A	36.55	A	40.45	F	49.62	F	51.37	F	55.44
Wuxi	A	35.27	A	32.55	A	40.82	A	43.64	A	46.27
Hangzhou	A	34.82	A	34.55	A	39.45	A	41.36	A	33.73
Shanghai	A	31.82	A	39.09	A	45.45	A	49.09	A	50.00
Yangzhou	A&F	25.46	A	42.82	H	42.75	G	47.68	F	118.60
Nanjing	A	15.00	A	37.36	A	35.64	A	43.18	A	39.82
Zhoushan	C	56.95	C	58.95	C	59.89	C	62.32	C	63.79
Xuancheng	C	56.11	C	57.89	H	57.14	G	61.79	H	81.95
Chizhou	C	52.74	C	54.42	H	58.24	G	70.10	H	96.93
Anqing	C	44.11	C	45.79	H	79.60	G	91.26	H	128.51
Ma’anshan	C	43.26	C	44.74	F	69.20	F	80.23	F	83.64
Chuzhou	C	42.63	C	44.11	G	58.79	G	93.99	H	127.06
Tongling	C	36.00	C	37.47	G	47.30	G	47.36	H	68.35
Yancheng	C	35.37	C	37.05	H	57.44	G	63.83	H	74.18
Huzhou	C	34.53	C	35.89	C	36.11	C	38.53	C	43.26
Wuhu	C	30.11	C	31.58	H	36.31	G	39.63	C	46.84
Taizhou	C	29.79	C	31.47	H	32.46	G	40.93	H	62.18
Changzhou	C	25.47	F	33.22	A	31.91	C	31.68	C	35.47
Shaoxing	C	25.26	B	27.89	F	30.93	F	36.34	C	37.89
Taizhou	F	56.57	F	55.22	F	45.93	F	52.13	F	60.22
Ningbo	F	56.32	F	56.83	F	45.74	F	56.76	F	58.75
Wenzhou	F	55.70	F	62.01	F	56.27	F	61.32	F	70.70
Jinhua	F	44.91	F	46.93	C	27.05	C	29.47	F	35.45
Nantong	H	20.89	H	30.88	H	29.42	G	36.01	H	43.26

The cities with the obstacle factor of the proportion of medical financial expenditure include Jiaxing, Hefei, Suzhou, Zhenjiang, Wuxi, Hangzhou, Shanghai, Yangzhou and Nanjing. Among them, Jiaxing has the highest overloading rate and Nanjing has the lowest overloading rate. The main reason for the relatively low proportion of financial expenditure on medical care is that cities, such as Shanghai and Nanjing, mainly spend their financial resources on education, social security and employment, and urban and rural communities. Alternatively, the city itself, such as Hefei, has a low level of economic development.

The cities with the obstacle factor of urban digital development index are Zhoushan, Xuancheng, Chizhou, Anqing, Ma ‘anshan, Chuzhou, Tongling, Yancheng, Wuhu, Taizhou, Changzhou and Shaoxing. Among them, Zhoushan has the highest overloading rate, and Shaoxing has the lowest overloading rate. The reason for the low urban digital development index is mainly the lack of digital transformation of urban industries or poor transformation results.

The cities with the obstacle factor of the number of beds are Taizhou, Ningbo, Wenzhou and Jinhua, among which Taizhou has the highest overload rate and Jinhua has the lowest overload rate. Nantong is the only city where the number of registered nurses is a key barrier. These two obstacles can be attributed to the poor supply level of medical resources, mainly due to the insufficient construction of urban medical facilities, medical materials and human resources construction failed to keep up with the population growth rate.

**Fig 8:**
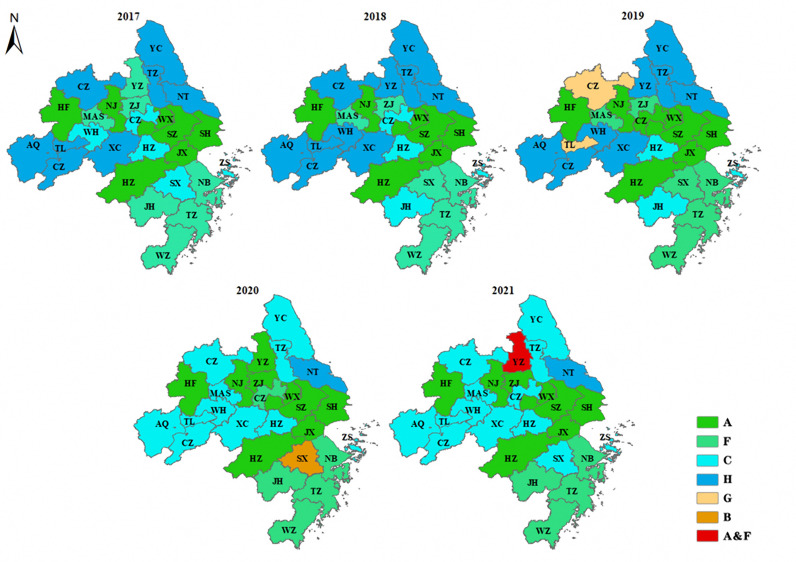
Changes in obstacles to UMSCC improvement from 2017-2021. These figures were created by the authors using a shapefile of the administrative division of China, which is publicly available on the government’s “National Geographic Information Public Service Platform of China” (Open data in China) web page at https://www.tianditu.gov.cn. The city name of abbreviation and raw data for these 27 cities are shown in Appendix 1 and 2.

As can be seen from Fig 8, there are 12 cities with no change in obstacle factors from 2017-2021, namely Jiaxing, Hefei, Suzhou, Wuxi, Hangzhou, Shanghai, Nanjing, Zhoushan, Huzhou, Taizhou, Ningbo and Wenzhou, but the overloading rates of obstacle factors in these cities has decreased. In addition, there are 10 cities with consistent changes in obstacle factors, all of which change from medical resource factors to the urban digital development index. These cities are Xuancheng, Chizhou, Anqing, Ma’anshan, Chuzhou, Tongling, Yancheng, Wuhu and Taizhou. The current level of digital technology has become the key factor for improving the carrying capacity of the medical system in these cities. The obstacle factors of Nantong in the past five years have varied between the number of practicing (assistant) physicians and the number of registered nurses, indicating that Nantong’s medical human resources are insufficient. The remaining four cities also experienced great changes. For example, Changzhou had the highest overloading rate in terms of the urban digital development index in 2017, 2018 and 2021, but it had the highest overloading rate in terms of the proportion of medical financial expenditure in 2019, and the highest overloading rate in terms of the number of beds in 2020.

## Discussion

### Recommendations

Based on the research results and analysis above, this paper puts forward some suggestions and recommendations to improve the public health system and rationalize the medical resource allocation for residents in the Yangtze River Delta region and provides references for the sustainable development of cities in the region.

(1) For cities where the key limiting factor is the proportion of medical financial expenditure, including Jiaxing, Hefei, Suzhou, Zhenjiang, Wuxi, Hangzhou, Shanghai, Yangzhou and Nanjing, the proportion of medical financial expenditure should be increased by adjusting the structure of financial expenditure or improving the economic level. For example, Shanghai should increase the proportion of medical expenditure through reasonable adjustment of the fiscal expenditure structure; Yangzhou, Zhenjiang and Hefei need to combine the industrial characteristics of each city to develop their economy, give full play to their geographical advantages, and change the low-benefit industrial pattern.
(2) For cities where the key limiting factor is the number of beds and the number of registered nurses, including Taizhou, Ningbo, Wenzhou, Jinhua and Nantong, mutual assistance relationships can be established with other cities through policy guidance. For example, Nantong currently has insufficient talent reserves, especially in terms of the number of registered nurses, and the existing human resources are difficult to meet the population demand in the next few years. Although the rapid growth of material resources can be promoted by increasing financial investment in the short term, the gap in health human resources is difficult to supplement in the short term. An integrated urban agglomeration smart network is encouraged to be constructed by establishing a high-speed transportation network and a cooperation platform for the co-construction and sharing of medical service resources and information, in order to eliminate the barriers in the provision and access of medical services among cities, and guide the sharing of high-quality medical resources. In terms of international cooperation, through the “Health Silk Road” (HSR), China will use the “Belt and Road” transport network - railways, ports, airports and logistics hubs - to provide medical and health assistance to partner countries [56], share quality medical resources, and improve the quality of medical services in partner countries.
(3) For cities where the key limiting factor is the urban digital development index, including Zhoushan, Xuancheng, Chizhou, Anqing, Ma ‘anshan, Chuzhou, Tongling, Yancheng, Wuhu, Taizhou, Changzhou and Shaoxing, the urban digital development index in the medical aspect can be improved through the combination of medical and digital. For example, the application of electronic health cards should be popularized, the promotion of multi-card collaborative applications should be accelerated, information sharing and business collaboration should be strengthened, and unified identification and certification services for residents should be provided. Moreover, the integration of emerging information technologies, such as blockchain, artificial intelligence, and edge computing, with the field of health should be promoted, and a collaborative development model driven by technology should be built, making “Internet of medical things” an important support for improving the efficiency of health service. The emergence of Internet of medical things brings many benefits to the future of digitalization, and this technology-based approach to treatment has enabled many countries to improve the quality and efficiency of treatment, improve patient well-being and government funding [57].

### Research Limitations

Although this study has achieved some interesting results, there are some limitations. For example, due to the under-developed of digital technology in the urban medical system in China, the data of the basic medical insurance participation rate and the urban digital development index from 2011-2016 are obtained mainly based on the 2017-2021 data, and the future research can make the results more effective by collecting the data over a longer period of time. In addition, at present, there is no definite index or model to measure the dynamic load quantificationally and accurately. With the frequent occurrence of urban disasters, more scholars should explore the dynamic load in the future.

## Conclusions

The purpose of this study is to analyze the UMSCC of 27 major cities in the Yangtze River Delta, and to provide a reference for other cities. The results are based on the panel data of 27 major cities in the YRDUA from 2011-2021 and the findings are as follows: (1)From the perspective of time, the UMSCC in the Yangtze River Delta shows an overall upward trend from 2011 to 2021, and the gap between the carrying capacity of the six types of urban medical systems is gradually narrowing. (2) From the perspective of space, the number of high-carrying cities and higher-carrying cities in the Yangtze River Delta region shows a gradual increasing trend from 2011 to 2021. (3) The center of gravity of UMSCC in the Yangtze River Delta is generally in the southwest direction and always located in Xuancheng, the middle of the Yangtze River Delta. (4) The obstacle factors of the UMSCC in the Yangtze River Delta are mainly concentrated in the proportion of medical financial expenditure, urban digital development index, the number of beds and the number of registered nurses.

In view of the obstacles to the UMSCC, this paper proposes three types of specific suggestions: the proportion of medical financial expenditure type, the medical resource type and the urban digital development index type. First, the proportion of medical expenditure can be increased by adjusting the structure of fiscal expenditure or improving the economic level. Second, through policy guidance, establishing mutual assistance relationships with other cities can improve the supply level of medical resources. And finally,the urban digital development index can be improved in the medical field through the combination of health care and digitalization.

## Supporting information

S1 Appendix 1 Raw data.
(XLSX)

S2 Appendix 2 City name of abbreviation.
(DOCX)
