# Supplementary material for: Spatio-temporal dynamics of urban medical system carrying capacity and their obstacle factors: A case study of Yangtze River Delta urban agglomeration
Source: PLoS One. 2025 Apr 9;20(4):e0319638. doi: 10.1371/journal.pone.0319638 (PMC11981656; doi:10.1371/journal.pone.0319638)
Supplement: S2 Appendix 2 — City name of abbreviation. (DOCX) [file pone.0319638.s002.docx]

Appendix 2. City name of abbreviation

| **Cities** | **Abbreviations** |
| --- | --- |
| Jiaxing | JX |
| Hefei | HF |
| Suzhou | SZ |
| Zhenjiang | ZJ |
| Wuxi | WX |
| Hangzhou | HZ |
| Shanghai | SH |
| Yangzhou | YZ |
| Nanjing | NJ |
| Zhoushan | ZS |
| Xuancheng | XC |
| Chizhou | CZ |
| Anqing | AQ |
| Ma'anshan | MAS |
| Chuzhou | CZ |
| Tongling | TL |
| Yancheng | YC |
| Huzhou | HZ |
| Wuhu | WH |
| Taizhou | TZ |
| Changzhou | CZ |
| Shaoxing | SX |
| Taizhou | TZ |
| Ningbo | NB |
| Wenzhou | WZ |
| Jinhua | JH |
| Nantong | NT |
